# Evaluating the health outcomes of aging in place: the role of medicaid aging waiver program on U.S. older adults

**DOI:** 10.1186/s12889-024-19498-3

**Published:** 2024-08-05

**Authors:** Xianhua Zai

**Affiliations:** 1 Max Planck Institute for Demographic Research, Konrad-Zuse-Straße 1, Rostock, Germany; 2 Max Planck – University of Helsinki Center for Social Inequalities in Population Health, Helsinki, Finland

**Keywords:** Medicaid aging waivers, Long-term care, Health, Aging in place, HRS

## Abstract

The Medicaid Aging Waiver program (MAW) subsidizes the cost of long-term care (LTC) at home or in communities to satisfy older people’s increasing desire to age in place. The MAW program might be health improving for older people by allowing them to age at home. However, less quality and quantity of home-based care comparing to nursing home care could offset some of the potential benefits. I use policy expenditure across states over time linked with detailed health information from the Health and Retirement Study (HRS) to identify the associated effects of MAWs on health outcomes of older adults who are at risk of needing LTC and who are resources constrained to be potentially eligible for Medicaid. Overall, the findings suggest that the MAW program is beneficial to health: a $1,000 increase in MAW spending for each older person results is associated with a 1.4 percent improvement in self-reported health status, a 1.5 percent reduction in functional mobility limitations, a 1.6 percent decrease in Instrumental Activities of Daily Living (IADL) limitations, and a 1.7 percent improvement in negative psychological feelings. For older people who are most likely not eligible for MAWs, such as those who are wealthy or in good health and do not require LTC, these health-improving effects have not been observed.

**Supplementary Information:**

The online version contains supplementary material available at 10.1186/s12889-024-19498-3.

## Introduction

The need for long-term care (LTC) in the United States has increased dramatically as its population ages [5, 29, 20, 24, 41]. To meet the elderly’s increasing demand for LTC without raising the government’s financial burden in covering the costly nursing home care services, the Medicaid Aging Waiver program (MAW) was implemented in the mid-1980s, and has been expanded its coverage rapidly since the early 1990s [27, 42]. The MAW provides exclusively Home and Community-Based Services (HCBS) to eligible older people who prefer to age in place and encourage them to delay entering nursing homes or rely less on nursing home care [1, 19, 37, 38, 48, 54].^1^

Despite its cost attractiveness, whether the expansion of MAWs improves health outcomes for the older population is unclear. On the one hand, a large body of literature demonstrates that aging-in-place can improve health by creating senses of belonging and self-control of lives, reducing feelings of loneliness, and facilitating social relationships [18, 40, 43, 44, 46, 49, 51, 53]. On the other hand, compared to nursing home care, there is typically less oversight of quality and quantity of home-based care [14, 26], which may reduce health outcomes for the affected population. For example, it has been shown that the training and skills of home-based care staff paid by MAWs are inadequate for particular groups, such as people with dementia, who are at risk of being inaccurately evaluated and given unsuitable care [8, 47]. In addition, older people who receive home-based care may have less contact with medical professionals than in a nursing home. In this case, some of their illnesses may go undiagnosed, even if their underlying health has deteriorated. Thus, the overall effect of MAWs on the health of older people demands detailed investigation.

This paper empirically examines how the MAW program is associated with the health outcomes for older Americans who are at the risk of needing LTC services and who are resources constrained to be potentially eligible for Medicaid. Using the restricted data of the Health and Retirement Study (HRS) and detailed data on state-level policy spending from 1998 to 2014, I demonstrate that the MAW benefits potentially eligible older people’s health in three dimensions. First, I show that a $1,000 increase in MAW expenditure for each older person decreases the probability of self-reporting poor health status by 1.4 percent. Second, I evaluate how the MAW program affect physical health. The findings show that a $1,000 increase in MAW generosity is associated with a 1.5 percent decrease in the probability of reporting mobility limitations and a 1.7 percent reduction in the likelihood of self-reporting Instrumental Activities of Daily Living (IADL) limitations. Third, the estimates also confirm that MAW generosity improves mental health by reducing the probability of reporting negative emotional feelings by 1.6 percent. To provide more evidence on the health-improving effect of MAWs, I run two placebo tests on wealthy older individuals who are implausibly eligible for Medicaid and on healthy older individuals who do not have LTC needs and find no association between MAW spending and health outcomes. In addition, the main estimates of MAWs on health for potentially qualified older people are robust to state-level socioeconomic controls and robust to different cutoffs of constructing the outcomes.

To shed light on how MAWs improve health for potentially eligible older people and for who are vulnerable of LTC needs, I provide suggestive evidence using a state-year panel of regressing MAW spending per capita on potential channels. I find that with the subsidized coverage from MAWs, an increase in generosity of the program significantly increases the likelihood to be helped by paid or unpaid helpers extensively or intensively. Specifically, a $1,000 increase in MAW spending per older person increases the likelihood to ever be helped by 9 percentage points, which is about 11 percent comparing to the outcome mean. In addition, the generosity of MAWs increases the likelihood to have at least one helper to help, to be helped at least one day, and to be helped at least one hour in the last month by approximately 10 percent. I also show that the estimate on using helpers paid out of pocket by old and low-income people is negative but statistically insignificant which is not surprising given that a smaller share of LTC needy people purchase any formal care from market. Further, I show that the MAW program seems to increase the chances of older people with LTC needs to utilize more healthcare services, in particular on going to the doctor and altering current healthy behaviors like cutting back on drinking and smoking.

### Related HCBS literature

The results are directly related to the research on how the HCBS program affects healthcare use and well-being of older people. Alecxih et al. [2] analyze Medicaid aged and physically disabled beneficiaries using negative binomial, Probit, and two-part model and find that HCBS usage is associated with fewer inpatient discharges, more outpatient service use, and mixed impacts on prescription drugs. Guo et al. [19]. use Medicaid claims data from the Cash and Counseling Demonstration and Evaluation (CCDE) program and an instrumental variable approach, finding that higher Medicaid home care spending significantly reduces both the likelihood and duration of nursing home use among elderly Medicaid enrollees. Miller [37] uses state-level data from 2000 to 2007 and applied multivariate fixed-effects models, showing that increased investment in HCBS is associated with reduced nursing home use for older adults but has no significant impact on use among working-age adults. Wang et al. [52] use Medicare and Medicaid data to investigate the impact of HCBS on nursing home placement for older adults with Alzheimer’s and related dementias and find that greater HCBS utilization is associated with a reduced likelihood of nursing home placement. Muramatsu et al. [38] also demonstrate that LTC systems are evolving towards greater support for community living, evidenced by higher functional impairment levels at admission and increased community discharge rates from nursing homes. Muramatsu et al. [39]. show that HCBS can mitigate depression risks associated with functional declines, particularly when informal support was lacking. Radke et al. [45] suggest that increased access to HCBS is associated with improved health outcomes and reduced overall Medicaid expenditures using state-level Medicaid and health surveys.

However, Konetzka et al. [31] use 2005 and 2012 Medicaid and Medicare data to examine the impact of HCBS on hospitalization rates among older, dual-eligible LTC users. It finds that HCBS users have a higher likelihood of hospitalization, with a 10 percentage point increase in overall admissions and a 3 percentage point increase in potentially avoidable admissions. Sands et al. [47] compare Medicaid recipients with dementia receiving LTC either through nursing homes or HCBS waivers. Using a longitudinal design, the researchers analyze monthly outcomes including inpatient and emergency department visits, as well as total Medicaid expenditures. Findings reveal that while waiver HCBS recipients have lower overall expenditures, they experience higher rates of inpatient admissions over time compared to nursing home residents. Wysocki et al. [55] use Medicaid and Medicare claims data from seven states to investigate hospitalization risks among elderly Medicaid LTC users transitioning from nursing homes to HCBS. Using Cox proportional hazards models and propensity score matching, they find that transitioners face a 40% higher risk of potentially preventable hospitalizations and a 58% higher risk of any hospitalization compared to stayers. To my best knowledge, this paper is the first to examine the extensive health effects of HCBS and thus adds to the discussion of benefits when assessing HCBS. I present convincing estimates by using the longitudinal HRS with a large representative sample of old people who are likely to require LTC in the United States linked with state-level demographic and economic variables that enable detailed robustness checks.

Second, the findings in this paper are broadly connected to the literature that estimates the benefits of public health policies. Studies of other Medicaid programs find that the Affordable Care Act (ACA) expansion improves self-reported health and the psychological health of low-income adults as well as infant health [10, 11, 17, 32, 36, 50]. Studies of Medicare show that Medicare benefits are associated with an improvement in self-reported health among older people [7, 30]. Studies of government welfare and nutrition programs also find an improvement in self-reported health status [4, 16, 21, 32]. The findings add to this line of research showing that government policy can effectively enhance people’s health conditions.

Third, the study is related to a smaller literature that evaluates the cost-effectiveness of HCBS. Many studies show that the HCBS program increases the overall Medicaid expenditure on LTC [18, 25, 28, 34]. The findings provide evidence that HCBS could save Medicaid health care spending by improving the health of older people. From a policy standpoint, the potential savings from health improvement justifies increasing investment in HCBS.

The paper is organized as follows. Section "Medicaid Aging Waivers" introduces the institutional background of HCBS and MAWs. Section "Data" describes the data, explains key health outcomes, and presents summary statistics. Section "Estimation" introduces the empirical model. Section "Results" reports the effects of MAWs on enrollment and a variety of health outcomes, explores mechanisms, and presents robustness checks. Section "Conclusion" concludes.

## Medicaid aging waivers

Medicaid Aging Waivers (MAWs) have been specifically designed to provide assistance to elderly individuals who would otherwise require nursing home care. The primary objective of these waivers is to enable old individuals to age gracefully in their own homes or communities, promoting their independence, well-being, and reducing the strain on long-term care (LTC) facilities. In the past, Medicaid coverage for LTC was limited to institutional settings like nursing homes, which resulted in a substantial increase in expenses. To address this issue and make LTC more affordable, the MAW program was introduced in the early 1980s. Its purpose is to curb the escalating costs associated with nursing home care by offering more affordable home or community-based alternatives (HCBS). The goal of MAWs is to provide care for older individuals with LTC needs in a familiar environment, enhancing their dignity in the aging process and improving their overall quality of life.

To qualify for MAWs, individuals must satisfy specific criteria, typically including being 65 years or older, being a resident of the state, having income and assets below certain thresholds, and demonstrating a need for LTC services that can be provided at home or in a community setting. Assessments of activities of daily living (ADLs) is often used as functional criteria to evaluate an individual’s ability to perform daily tasks. These assessments help determine the required level of care and eligibility for MAWs. The exact eligibility requirements vary from state to state. In 2018, 40 states set their annual income thresholds at 300 percent of the Supplemental Security Income (SSI) level (equivalent to $27,000 for a single individual), 8 states set it between 100 and 300 percent of SSI (ranging from $9,000 to $27,000), and 3 states used 100 percent of SSI ($9,000). Regarding asset limits, 39 states set it at $2,000, 6 states had no asset limit, 8 percent of states set it between $2,500 and $4,000, and 2 states used $1,600 in 2018. In section "Sample selection", I select the working sample with individuals who are at risk of requiring LTC and being covered by MAWs based on the age, ADL, and constraint of resources of old individuals.

**Fig. 1:**
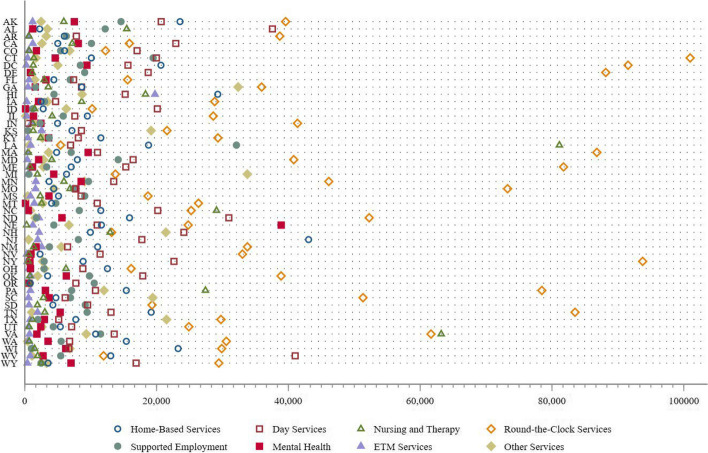
Variation in spending per enrollee for each service covered under MAWs in 2014

Notes: The plot displays the variation in spending per enrollee for each service covered under MAWs in 2014 across states. The x-axis is the dollars spent per participant. The y-axis is the abbreviation of each state.

There are several advantages offered by MAWs that are beneficial for health. Firstly, they grant each state the flexibility and autonomy to determine services that promote the participants’ health. These services typically encompass personal care, home health care, day care, and home modifications, although the extent of coverage may differ among states. In 2018, home-based services were provided in 43 states, nursing or therapy services in 36 states, equipment and technology modifications (ETM) in 39 states, round-the-clock services in 20 states, day services in 31 states, and case management services in 32 states. By tailoring the services provided, individual states can address the specific needs of their aging populations, thereby allowing for greater flexibility in delivering LTC services. Moreover, MAWs foster innovation by enabling states to explore inventive approaches to LTC delivery.^2^ As illustrated in Figure 1, the spending levels on different services covered by MAWs varied significantly between states in 2014. For instance, Oregon allocated only $826 per participant for home- based services, whereas New Jersey expended $43,066 per participant.

Secondly, the implementation of MAWs involves a collaborative effort among state governments, healthcare providers, and community organizations. The process begins with the state government submitting a MAW application to CMS, which outlines the program’s services, eligibility requirements, participant limits, and associated costs. CMS evaluates the application based on criteria such as financial feasibility, cost-effectiveness, and quality of care. Once the MAW program receives approval, the implementation process includes enrolling participants, assessing their needs, developing personalized care plans, and delivering home-based services and support. Enrollment of eligible older adults is handled by the state health agency, while healthcare providers like home health agencies and adult day care centers offer LTC services to participants. Community organizations, such as non-profit agencies and advocacy groups, may also provide support and resources to participants and their families. Additionally, CMS regularly monitors the MAW program to ensure compliance with the terms of the waiver agreement. The state is required to submit periodic reports on the program’s performance and quality of care.

**Fig. 2:**
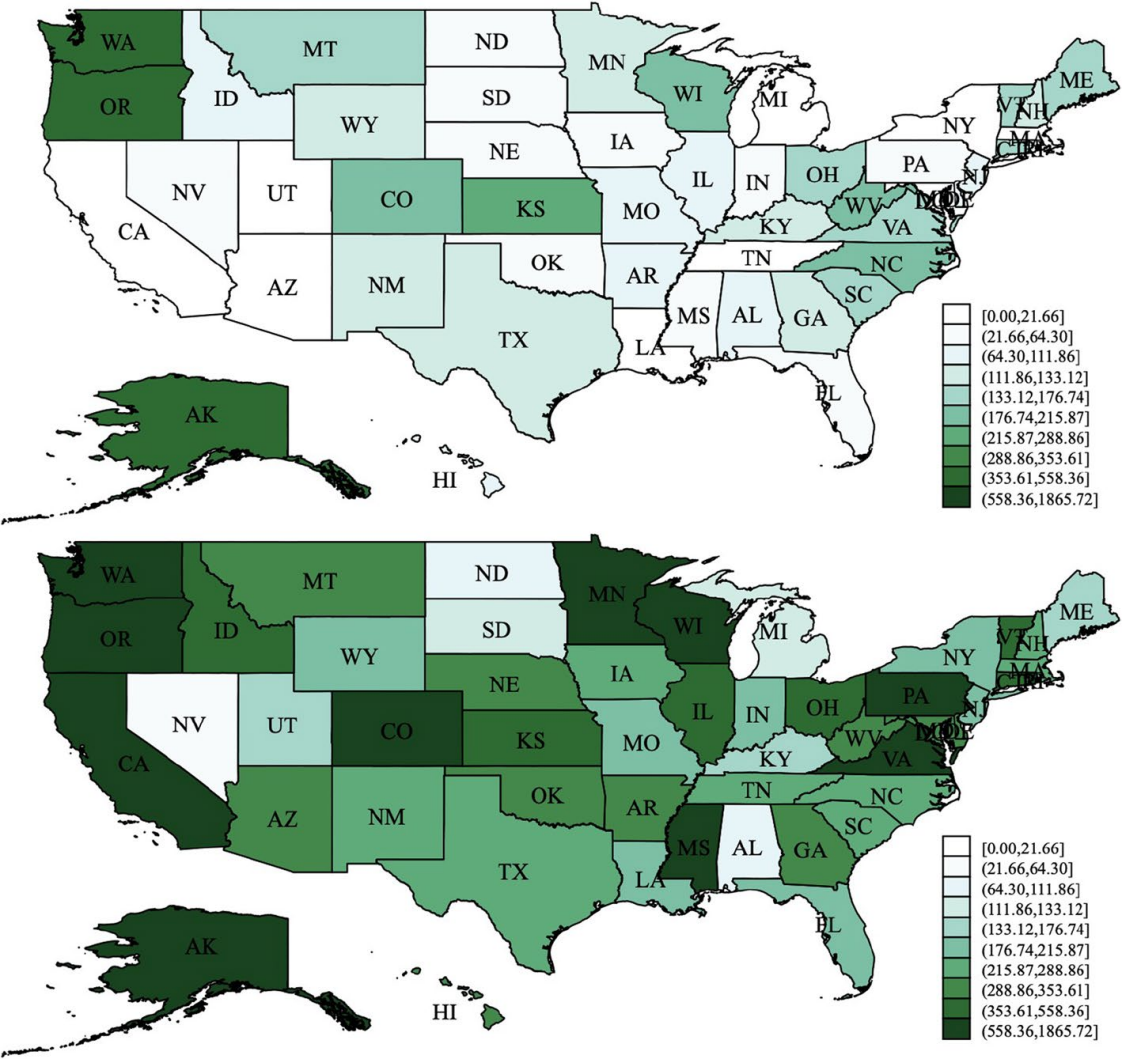
State variation in MAW expenditure per older person in 1998 and 2014 Notes: Bins represent the deciles of the distribution for the state’s average MAW spending for people aged over 65 in 1998 and 2014. The darker the color on the map, the higher the per capita MAW spending in the state

The distinct characteristics and implementation procedures of MAWs grant states the freedom to tailor their programs according to the specific requirements of their aging populations. Consequently, there is notable diversity among MAW programs across states, encompassing variations in offered services, eligibility criteria, methods of delivery and payment, as well as practices for monitoring quality. Figure 2 illustrates the considerable disparity in MAW expenditures ($2014) among all 50 states in the United States during the 1998-2014 period, which can be attributed to differences in policy design. More details on MAW spending for each state can be referred to Liu and Zai [35].

## Data

The first data source is the MAW policy information for older adults from 1996 to 2014 from the Centers for Medicare & Medicaid Services (CMS).^3^ It includes annual reports of expenditures and enrollment in MAWs for each state.^4^ The main independent variable is the MAW expenditure per capita in constant 2014 dollars, which is calculated using the population aged 65 and older at each state.

The second data source is the Health and Retirement Study (HRS), a longitudinal survey representative of Americans aged 51 and older which began in 1992 and surveys every other year. The survey administrators send out a detailed questionnaire in person or via telephone that collects respondents’ information regarding their demographic characteristics, detailed health outcomes, and their financial circumstances, including incomes and assets. The restricted-access HRS includes the state of residence for each respondent, which is used to merge with the MAW policy information at the state level.

Since the changes in MAW spending within states might be correlated with state-level confounders, that also affect health outcomes, I also supplement with other data to test the robustness of the results. Specifically, the Bureau of Labor Statistics (BLS) provides information about state-level unemployment rates and employment rates; the Bureau of Economic Analysis (BEA) Regional Economic Accounts presents information about GDP, personal income (PI), and personal consumption expenditure (PCE); the Census Bureau supplies information on demographic characteristics of states;^5^ and the MIT Election Lab includes information on the political affiliation of state governors.^6^ The state-level demographic and economic variables are employed to test the sensitivity of our results in section "Results".

### Sample selection

To estimate the health effect of MAWs, I restrict the HRS working sample to those who are potentially in need of LTC and whose resources might qualify them for Medicaid. First, I select respondents from the HRS survey who are 65 and above, which is the cutoff age for MAWs. Second, I restrict the sample to HRS respondents who have at least two items of difficulty in activities of daily living (ADL) such as bathing, eating, dressing, getting in or out of bed, and walking across a room. Thus, these old people selected are mostly likely in need of LTC services. Third, I only include respondents to the sample who are resources constrained with an income ($2014) less than 138 percent FPL or with an asset value ($2014) lower than 4,000 for a family of two or with no more than 12 education years.^7^ The resulting working sample includes around 3,000 unique individuals with 9,300 observations from 1998 to 2014.^8^

### Key variables

I use information about the detailed health outcomes to estimate how MAWs affect the well- being of older people. The HRS asks respondents to self-report their general health status where 1 is for excellent, 2 for very good, 3 for good, 4 for fair, and 5 for poor. We create a subjective health indicator, which equals 1 if the self-reported health is fair or poor, and 0 otherwise.^9^ While the self-reported health might be subjective and prone to recall errors, it has been proved to be a good predictor of health outcomes [13, 22, 32]. I provide estimates from alternative health indicators as robustness checks.

I also use objective measures on physical health conditions. The HRS has detailed information about functional limitations. Specifically, the mobility difficulty index refers to whether respondents have any problem in walking 1 block, walking several blocks, walking across a room, climbing 1 flight of stairs, and climbing several flights of stairs. The HRS also provides an index about IADLs, which assesses difficulties in using the phone, managing money, taking medications, shopping for groceries, and preparing hot meals. These mobility and IADL indexes all range from 0 to 5. For example, an index with a value of 5 means that an individual has difficulties with all of the functional limitations, while a value of 0 means that the individual has no issues related to physical health. I create two dichotomous indicators that equal one if an individual has a certain number of limitations and zero, otherwise for mobility and IADL difficulty, respectively. Section "Results" reports the main estimates using the mobility difficulty indicator with at least 2 items of limitations and using the IADL difficulty indicator with at least 1 item of limitations. The results on other indicators with different cutoffs are also reported in section "Results" as robustness checks.^10^

I further use information about mental health to evaluate the impact of MAWs on depression. The HRS asks respondents about their mental health using the Center for Epidemiologic Studies Depression (CESD) score. The CESD score captures the number of negative sentiments experienced by the respondent most of the time in the past two years including, whether the individual was depressed, felt alone, felt sad, had restless sleep, felt everything was an effort, could not get going, felt unhappy, and did not enjoy life. The CESD scale has been validated to identify major depression in older adults [23]. I create a depressed indicator that equals 1 if an individual reported at least 1 item of negative feelings and 0 otherwise. I also show estimates from other depressed indicators in section "Results".

### Sample statistics

Table 1 presents the summary statistics of the working sample – HRS respondents who are aged 65 and older with LTC needs and are potentially eligible for MAWs. About 70 percent of these individuals are female since women in general live longer than men. The average educational years of these respondents are around 10. On average, each individual has about two siblings. The majority of respondents are white (74 percent), and 21 percent are black. The average age of respondents is about 80 years. Approximately 36 percent of respondents are married or live with a partner and 51 percent have lost their spouse or partner. For health outcomes, on average, individuals assess their health status as fair and around 76 percent report their health as fair or poor. In the meantime, about 92 percent of the sample report having at least 2 items of mobility limitations and the average limitations on mobility is about 4 items. The average number of items in IADL limitation is about 3 and 82 percent of older individuals with LTC needs have at least 1 item in IADL limitations. The average CESD depression score is 3.4 out of 8 and majority of individuals have at least one negative emotion.

**Table 1:** Summary statistics of the working sample

Variable	Mean	S.D	Unique individuals	Obs
***Time-invariant demographics***				
Female	0.70	0.46	3,124	9,322
Education years	9.92	3.88	3,124	9,322
Number of siblings	2.15	2.31	3,124	9,322
Race/ethnicity				
White	0.74	0.44	3,122	9,316
Black/African	0.21	0.41	3,122	9,316
Other	0.05	0.21	3,122	9,316
***Time-varying demographics***				
Age	80.28	8.59	3,124	9,322
Marital status				
Married/partnered	0.36	0.48	3,124	9,322
Separated/divorced	0.10	0.30	3,124	9,322
Widowed	0.51	0.50	3,124	9,322
Never married	0.03	0.18	3,124	9,322
***Heath variables***				
Self-reported health (1–5)	4.09	0.95	3,124	9,305
Poor or fair health indicator (0/1)	0.76	0.43	3,124	9,305
Some mobility limitations (0–5)	3.93	1.35	3,124	9,283
Mobility limitation indicator (0/1)	0.92	0.27	3,124	9,283
Some IADL limitations (0–5)	2.56	1.8	3,124	9,319
IADL limitation indicator (0/1)	0.82	0.38	3,124	9,319
CESD scores (0–8)	3.38	2.36	2,332	5,975
Depression indicator (0/1)	0.89	0.32	2,332	5,975

Notes: The data used are from HRS 1998 to 2014 of individuals who are potentially in need of LTC and eligible for MAWs. The definitions of these variables can be found in text of section "Data"

## Estimation

I estimate the associated health effects of MAWs among the vulnerable older population who are at risk of requiring LTC with the following specification:

1 $${Y}_{ist} = {\alpha }_{0} + \delta MA{W}_{s\overline{t} }+ {\alpha }_{i} + {\mu }_{t} + {\eta }_{s} \times t + {X}_{ist}\beta +{\epsilon }_{ist}$$

where *Y*_*ist*_ is the health outcome of an individual *i* in state *s* surveyed in year *t*. *MAW*_*st*_¯ is the average expenditure of MAWs per person in state *s* in year *t* and *t −* 1. For example, the health outcome of each individual in survey year 2000 is regressed on MAW expenditure averaged in 1999 and 2000. The construction of the policy variable takes into account that the HRS survey is conducted every two years and the health outcomes of the sample is a function of average MAW generosity in the current year and in the previous year. The individual fixed effect, *α*_*i*_, controls for the unobservable individual-level time invariant factors such as preference of health-keeping, health or exercise habits. The year fixed effect, *µ*_*t*_, controls for common temporal shocks across states that could affect health outcomes. *η*_*s*_ × *t* is the state-specific linear time trend, which controls for the heterogeneous trends in health across states. *X*_*ist*_ is a vector of time-varying demographic characteristics of individuals, such as age, age squared, the number of living siblings, and marital status. The standard errors are clustered at the state level.

The coefficient of interest, *δ*, measures the impact of MAW spending on individuals’ health outcomes. As suggested by Callaway et al. [6], two assumptions are needed to interpret this coefficient as causal. First, it is assumed that states with higher MAW expenditure in 1998 did not expand their spending differently for non-MAW-expansion- related reasons. That is, states with different MAW expenditure in 1998 are on parallel pre- expansion trends. Second, it is assumed that older people with LTC needs who are potentially eligible for the program in each state receive the same treatment effect for an extra dollar spent, even if they are not paid the same amount initially. That is, it is assumed homogeneous treatment effects across states. These assumptions are tested in section "Threats to identification". In addition, I estimate equation (1) with the most demanding specification that includes individual fixed effects. This specification takes advantage of the within-individual variation in MAW generosity across years that may come from MAW spending change in their state of residence or cross- state migration.

### Threats to identification

The main identification assumption relies on that the within-state variation of HCBS generosity over years is not correlated with other unobservable confounders that might also affect the health outcomes of interest. One might be concerned that states chose HCBS expansion during the 1990s based on the health status of their residents. For example, states could expand HCBS if health or other related well-being factors of older adults were worse or if they think home or community-setting can benefit older adults more on health. One might also be worried that individuals changed health-related behavior in anticipation of the HCBS expansion. To mitigate these possible concerns, the effect of pre-expansion HCBS spending in 1998 on a range of health-related outcomes for the period 1992-1998 is estimated in a simple linear regression [3].

2 $$y_{i s t}= \alpha + \beta_0 {HCBS}_{s}^{1998} + \beta_1 {HCBS}_{s}^{1998} \times (y-y^{1998}) + \xi_{st}$$

where *y* is a series of dependent variables to be tested against the HCBS spending per older person in 1998 when the program expansion began. I test for balance in levels (*H*_0_ : *β*_0_ = 0) in 1998 and in linear pre-1998 trends (*H*_0_ : *β*_1_ = 0).

Appendix Table A1 demonstrates the estimates of the per capita HCBS generosity in 1998 on a battery of health-related outcomes. Column 1 reports the estimates in a simple OLS without any controls. Column 2 adds the controls in the baseline specification (1). Panel A tests the relationship between initial HCBS generosity on health outcomes of interest in the paper and Panel B further shows the tests on related health care use outcomes. The generosity of HCBS is negatively correlated with the probability of having poor health and positively correlated with the probability of visiting a dentist in a simple univariate regression. The signs of these coefficients change after controlling for demographics of individuals and become statistically insignificant except for the coefficient on mobility limitations. However, the coefficients on all health outcomes are not consistently positive or negative, which supports that there is no systematic correlation between the HCBS expansion and health outcomes of interest. Likely, the variation of HCBS is more to be driven by institutional features rather than economic or social environment.

One might also be worried that HCBS generosity is correlated with the economic condition, which in turn impacts individual health. To address this issue, I construct a state-year panel from 1999 to 2014 using different sources of economic variables such as unemployment rate, employment rate, GDP per capita, personal income (PI) per capita, and personal consumption expenditure (PCE) per capita. These state economic measures are regressed on the HCBS spending controlling for state and year fixed effects. I allow flexible functional form of these economic variables and the results are reported in Appendix Table A2. For the first four columns, I use flexible functions of unemployment rate and employment rate. I then further add different income and consumption variables.

Overall, the results show that state-specific economic variables are not correlated with HCBS. The employment rate is positively related to HCBS and the unemployment rate is negatively related to HCBS, in columns 1 and 3. These relationships, however, are not statistically significant once allowing quadratic or cubic form and adding in more economic controls such as income and expenditure. One might also worry that the HCBS size could be correlated with lagged economic conditions. For example, if states experienced high unemployment rates, the size of HCBS for older population could be decreased if state legislators are constrained by fiscal resources. Appendix Table A3 reports the results of lagged economic conditions on HCBS spending. As predicted in column 1, states with high unemployment rate in the last year have less HCBS spending and the estimate is statistically significant. When I further allow flexible unemployment rate format and add more state-level economic controls, the relationship between lagged economic factors and HCBS generosity becomes nonexistent. Nonetheless, I check the sensitivity of our results after controlling for state-level factors in section "Robustness".

Another possible concern could be that the health change of older individuals might be driven by other contemporaneous social programs. I use the detailed consumer spending expenditure from Bureau Economic Analysis on health-related products to address this concern. Specifically, I explore the relationship between HCBS generosity and health care spending, net health insurance spending, and life insurance spending which are mostly relevant to health of the older population. Appendix Table A4 shows the estimates of the respective spending in each column. All specifications control for state-economic factors including unemployment/employment rate, income, and expenditure in each state. The relationships of HCBS with other health care spending, health insurance and life insurance spending are not obvious and statistically insignificant, which is assuring to the results.

## Results

In this section, I show how MAW spending is associated with enrollment in this program. Then, I estimate the association between MAWs and health outcomes among the older population who require LTC needs and who are potentially qualified for Medicaid.

### MAW spending and enrollment

**Fig. 3:**
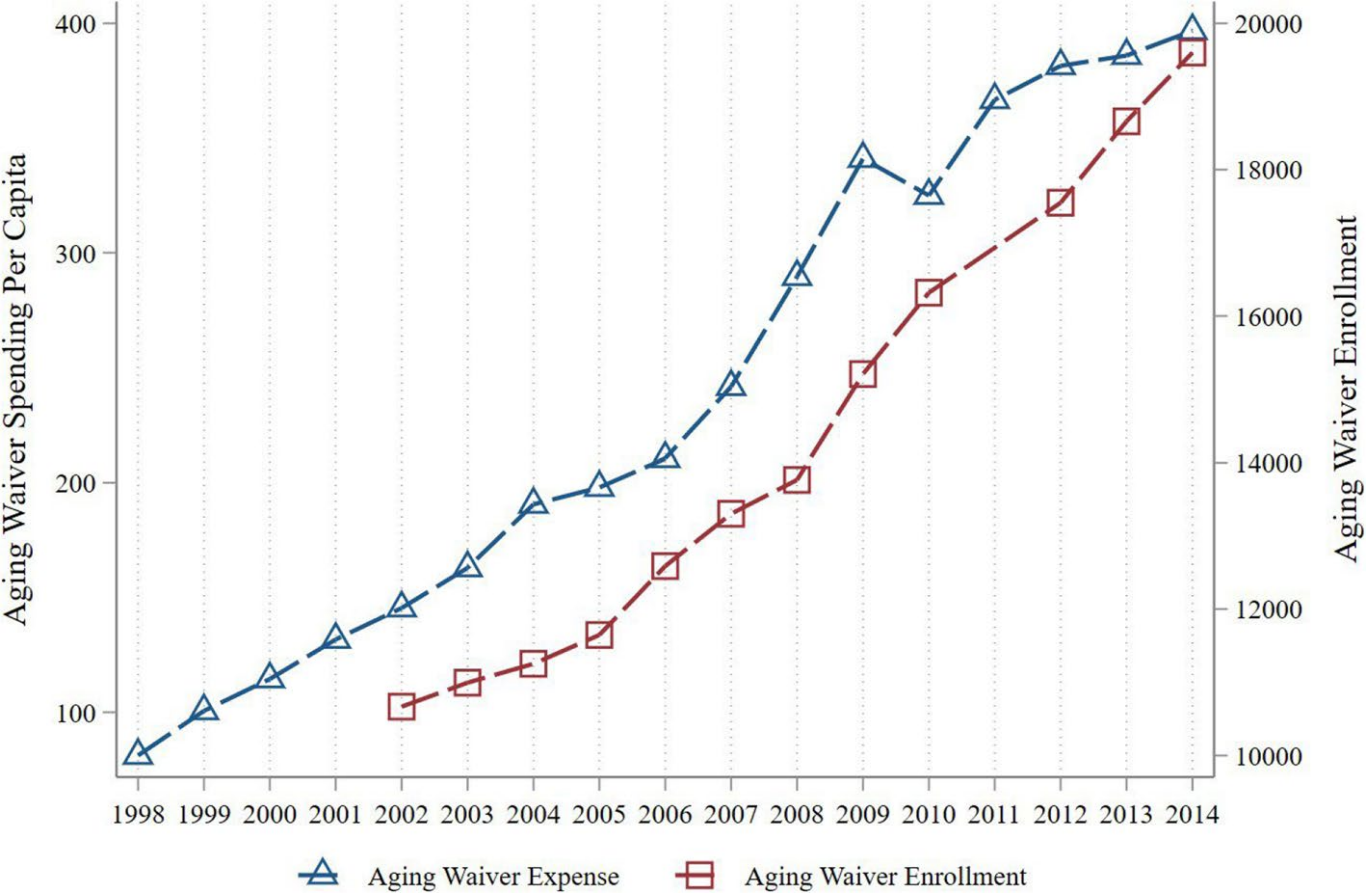
MAW spending ($2014) and enrollment from 1998 to 2014 Notes: The data used are drawn from CMS data on enrollment in the MAW programs. The plot shows the spending per capita ($2014) on and the enrollment in MAWs over the 1998 to 2014 period. The left y-axis corresponds to spending and the right y-axis corresponds to enrollment

Figure 3 depicts MAW spending and its enrollment using the policy data from 1998 to 2014. The trend of enrollment in the MAW program is almost parallel with its expenditure despite a slight decline in spending in 2010.

As discussed in section "Medicaid aging waivers", states have discretion to allocate MAW resources. To estimate the effect of MAWs on enrollment, I regress the program spending on its enrollment after controlling for state and year fixed effects. Table 2 reports the estimates of the impact of MAW spending on enrollment with different specifications in each column. Column 1 shows the estimate for the specification with only state and year fixed effects. Column 2 adds additional demographic controls at the state level such as poverty, education, percentage white, and percentage married. Column 3 further includes state-level economic conditions such as unemployment rate and employment rate. Column 4 adds GDP per capita ($2014), personal income per capita ($2014), and personal consumption per capita ($2014) to control for economic changes at the state level. Column 5 adds lagged unemployment rate, lagged GDP per capita ($2014), lagged personal consumption per capita ($2014), together with their respective quadratic and cubic forms to flexibly reflect any shocks to the economy in each state. Overall, one dollar increase in MAW spending per person is correlated with an increase in the program enrollment by 8 to 13. With a mean of MAW spending per person at $240, the increase in the corresponding enrollment is about 13.6 to 22.1 percent increase relative to the outcome mean. The estimates are robust with different demographic and flexible economic controls at the state level.

**Table 2:** The effect of MAW spending on enrollment

Dependent Variable: Enrollment in MAWs					
MAW expenditures per person	(1)	(2)	(3)	(4)	(5)
	8.099	8.170	8.267	9.580	13.038*
	(10.745)	(9.132)	(8.973)	(8.684)	(7.249)
Mean of dependent variable	14,163	14,113	14,113	14,113	14,113
Observations	576	573	573	573	573
Adjusted R-squared	0.82	0.84	0.84	0.84	0.86
State + Year FE	Y	Y	Y	Y	Y
Demographics		Y	Y	Y	Y
Employment rate			Y	Y	Y
GDP and consumption				Y	Y
Lag economic conditions					Y

Notes: The data used are from CMS about enrollment in the MAW program from 1998 to 2014. Each cell reports estimates from a separate specification, weighted by state populations. Column 1 shows the simple estimate only with state and year fixed effects. Column 2 adds demographic variables at the state level such as poverty, education, percentage white, and percentage married. Column 3 further includes unemployment rate and employment rate to account for state-level economic conditions. Column 4 adds GDP per capita ($2014), personal income per capita ($2014), and personal consumption per capita ($2014) to further control for economic changes at the state level. Column 5 adds lagged economic conditions on unemployment rate, GDP per capita ($2014), personal consumption per capita ($2014) as well as its quadratic and cubic forms to flexibly reflect any shocks of economic situation in each state. The standard errors are clustered by state

*p < 0.10

### Results of MAWs on health

Table 3 reports the estimates of MAWs on health outcomes using the equation (1). Specifically, a $1,000 increase in MAW spending per person ($2014) is significantly associated with an approximate 1.1 percentage point decrease in the probability of self-reporting poor health status (column 1). Relative to the outcome mean of our working sample, 0.76, the estimated effect size corresponds to a reduction in the probability of reporting poor health by 1.4 percent. Column 2 shows that the MAW program generosity is negatively correlated with the probability of individuals having mobility limitations. The improvement in mobility-related health is about 1.4 percentage points with statistical significance, which is approximately 1.5 percent relative to the outcome mean at 0.92, given a $1,000 increase in MAW spending. Similarly, MAWs significantly improve the likelihood of reporting less IADL limitations by 1.4 percentage points, which is about 1.7 percent reduction with an $1,000 increase in MAW generosity per older person (column 3). Column 4 reports that the MAW program is beneficial to improve mental health. A $1,000 increase in MAW spending per person decreases the probability of reporting negative emotional feelings by 1.5 percentage points (1.6 percent relative to the outcome mean). Appendix Table A5 reports the effects of MAWs on each health outcome with other cutoffs in Panels A-D, respectively. Despite some of these alternative construction of health outcomes lose statistical significance, all of the signs of the results maintain negative.^11^ In addition, we estimate how the MAW program reduces morbidity events such as cancer, lung disease, heart disease, and strokes and do not find any significant reeducation in these episodes although the signs are negative (Appendix Table A6).

**Table 3:** The effect of MAWs on health outcomes

	(1)	(2)	(3)	(4)
	Poor health	Mobility limitation	IADL limitation	Depression
MAW expenditures per person ($1,000)	-0.109*	-0.141***	-0.141***	-0.145***
	(0.061)	(0.035)	(0.041)	(0.053)
Mean of dependent variable	0.76	0.92	0.82	0.89
Number of individuals	3,122	3,109	3,122	1,902
Observations	9,303	9,268	9,317	5,545
Adjusted R-squared	0.31	0.20	0.32	0.30

Notes: The data used are from the HRS from 1998 to 2014 of individuals who are potentially in need of LTC and eligible for MAWs. Each cell reports estimates using Eq. (1) for each dependent variable. Poor health is an indicator showing that an individual self-assesses his or her general health status as fair or poor. Mobility limitation is an indicator of having at least 2 items of limitations in walking 1 block, walking several blocks, walking across a room, climbing 1 flight of stairs, and climbing several flights of stairs. IADL limitation is an indicator of having at least 1 item of limitations in using the phone, managing money, taking medications, shopping for groceries, and preparing hot meals. Depression is an indicator of feeling at least 1 negative emotions in the past two years such as felt depressed, felt alone, felt sad, had restless sleep, felt everything was an effort, could not get going, felt unhappy, and did not enjoy life. All models control for individual FEs, year FEs, state-specific linear time trends, and demographics of individuals such as age, age squared, marital status, and number of siblings. Standard errors are clustered at the state level

***p < 0.01, *p < 0.10

### Robustness

In this section, I report some robustness checks on the estimates of the effect of MAWs on health for potentially LTC needy and Medicaid qualified older people. First, I run two placebo tests to provide supportive evidence that MAWs indeed have beneficial effects on older people’s health. As introduced in the background section "Medicaid aging waivers", having constrained resources is a requirement to be eligible for MAWs. For those who have a sizable income or assets, MAWs are not supposed to have effects on their health. Table 4 presents supportive estimates that MAWs do not affect the health outcomes for older people who have incomes ($2014) larger than 95 percentile of the distribution or who have assets with values ($2014) larger than 50,000. In addition, for those who are younger and do not have ADL limitations, they are not eligible for MAWs such that the program should not affect their health. Table 5 shows estimates of MAWs on health among people who are less than 65 years old and who are free from any ADL limitations using the specification (1). All estimates are positive and the estimate on mobility limitation is statistically significant, which supports the main results that MAWs improve health for people who are potentially qualified for Medicaid and who are at risk of requiring LTC services.

**Table 4:** Placebo tests of the results of MAWs on health for the resources generous sample

	(1)	(2)	(3)	(4)
	Poor health	Mobility limitation	IADL limitation	Depression
MAW expenditures per person ($1,000)	-0.011	0.003	-0.008	-0.003
	(0.013)	(0.011)	(0.010)	(0.019)
Mean of dependent variable	0.17	0.20	0.09	0.45
Number of individuals	15,426	15,411	15,412	14,533
Observations	81,941	81,910	81,908	76,260
Adjusted R-squared	0.45	0.49	0.41	0.35

Notes: The data used are from the HRS from 1998 to 2014 of individuals who have incomes larger than 95 percentile of the distribution or who have assets value larger than $50,000. Each cell reports estimates using Eq. (1) for each dependent variable. Poor health is an indicator showing that an individual self-assesses his or her general health status as fair or poor. Mobility limitation is an indicator of having at least 2 items of limitations in walking 1 block, walking several blocks, walking across a room, climbing 1 flight of stairs, and climbing several flights of stairs. IADL limitation is an indicator of having at least 1 item of limitations in using the phone, managing money, taking medications, shopping for groceries, and preparing hot meals. Depression is an indicator of feeling at least 1 negative emotions in the past two years such as felt depressed, felt alone, felt sad, had restless sleep, felt everything was an effort, could not get going, felt unhappy, and did not enjoy life. All models control for individual FEs, year FEs, state-specific linear time trends, and demographics of individuals such as age, age squared, marital status, and number of siblings. Standard errors are clustered at the state level

**Table 5:** Placebo tests of the results of MAWs on health for the healthy sample

	(1)	(2)	(3)	(4)
	Poor health	Mobility limitation	IADL limitation	Depression
MAW expenditure per person ($1,000)	0.003 (0.020)	0.022* (0.011)	0.014 (0.010)	0.013 (0.023)
Mean of dependent variable	0.18	0.13	0.04	0.51
Number of individuals	18,230	18,236	18,235	17,392
Observations	72,416	72,446	72,435	68,370
Adjusted R-squared	0.50	0.45	0.30	0.37

Notes:The data used are from the HRS from 1998 to 2014 of individuals who are younger than 65 and who have no limitations in ADL. Each cell reports estimates using Eq. (1) for each dependent variable. Poor health is an indicator showing that an individual self-assesses his or her general health status as fair or poor. Mobility limitation is an indicator of having at least 2 items of limitations in walking 1 block, walking several blocks, walking across a room, climbing 1 flight of stairs, and climbing several flights of stairs. IADL limitation is an indicator of having at least 1 item of limitations in using the phone, managing money, taking medications, shopping for groceries, and preparing hot meals. Depression is an indicator of feeling at least 1 negative emotions in the past two years such as felt depressed, felt alone, felt sad, had restless sleep, felt everything was an effort, could not get going, felt unhappy, and did not enjoy life. All models control for individual FEs, year FEs, state-specific linear time trends, and demographics of individuals such as age, age squared, marital status, and number of siblings. Standard errors are clustered at the state level

*p < 0.10

Second, one might be concerned that our results might be biased if the state-level characteristics correlate with MAW spending. To address this issue, Appendix Table A7 reports the results after controlling for state-level socioeconomic variables such as percentage below the poverty level, education level, percentage female, percentage white, percentage married, personal income per capita, employment rate, and political affiliation of the governor using the model (1). Overall, the health estimates are robust across specifications with state- level controls. If anything, the magnitude of the health improvement by the MAW program is larger with state-level controls.

Third, the main identification variation comes from changes of MAW expenditures over years within individuals. However, one might be concerned that the endogenous migration of individuals who move across states could bias the results. For example, individuals who anticipate LTC needs and are possibly eligible for Medicaid would move to states with more generous MAWs if they were more self-aware of their health conditions or they value health more than others. To address this concern, I limit the sample of never-movers and find assuring results that are quite robust in Appendix Table A8.

### Channels on health improvement by MAWs

To shed light on how MAWs are beneficial for health of the aging population who are at risk of needing LTC services, I explore potential channels through which the MAW program improves health outcomes. For one, MAWs subsidize professional providers to offer in-home services to the eligible, thus increasing the probability to have helpers. In addition, MAWs increase the chances to expose with healthcare providers who often give advice on healthy behaviors and on consumption of healthcare services. Taking advantage of the detailed questions asked in the HRS, Tables 6, 7 and 8 present preliminary and supportive evidence that MAWs indeed allow older people with LTC needs to age in place.^12^

**Table 6:** Channel of MAWs on the availability of helpers

	(1)	(2)	(3)	(4)	(5)
		Helped last month			
	Any helper ever	Any helper	Any day	Any hour	Any paid helper
MAW expenditures per person ($1,000)	0.089**	0.081*	0.083**	0.079*	-0.011
	(0.040)	(0.041)	(0.041)	(0.044)	(0.044)
Mean of dependent variable	0.79	0.77	0.77	0.76	0.28
Observations	10,307	10,280	10,230	9,726	7,911

Notes: The data used are from the HRS 1998 to 2014 of individuals who are potentially in need of LTC and eligible for MAWs. Each cell reports estimates using a TWFE model with state and year FEs for each dependent variable. Any helper ever in column 1 is an indicator showing that an individual had at least one helper who ever helped them. Columns 2–5 indicate the help status since the last month. Any helper in column 2 represents whether there was any helper helped in the last month; any day in column 3 shows whether there was any day got helped from anyone in the last month; any hour in column 4 indicates whether there was any hour got helped from anyone in the last month; and any paid helper in column 5 shows whether an individual paid to get helped in the last month. All models control for state FEs, year FEs, and state-specific linear time trends. Standard errors are clustered at the state level

**p < 0.05, *p < 0.10

Table 6 shows the channel of MAWs on the availability of helpers that help vulnerable older people with LTC needs. Column 1 reports that a $1,000 increase in MAW spending per person increases the likelihood to ever be helped by either paid or unpaid helpers by 9 percentage points, which is about 11 percent comparing to the outcome mean. Columns 2 to 5 further present the estimates of the availability of helpers helped last month since the interview date. The results in columns 2 to 4 suggest that the generosity of MAWs significantly increases the likelihood of having at least one helper to help, of being helped at least one day, and of being helped at least one hour in the last month by approximately 10 percent. The estimate on using helpers paid out of pocket by individuals in column 5 is negative but statistically insignificant which is not surprising given that a smaller share of LTC needy people purchase any formal care from market.

Table 7 further shows the channel of MAWs on the probability of using healthcare services. The MAW program seems to increase the chances of older people with LTC needs to consume more healthcare. The estimate on increasing the number of doctor visits is statistically significant: a $1,000 increase in MAW generosity increases the times to pay doctor visits by 10 times, an approximate 56 percent increase from the outcome mean. The estimates on other outcomes such as paid home health care out of pocket, hospital use, and medication use are positive but indistinguishable from zero.

**Table 7:** Channel of MAWs on the probability of using healthcare services

	(1)	(2)	(3)	(4)	(5)
	Home health use	Hospital use	Medication	Hospital overnight	N. of doctor visits
MAW expenditures	0.028	0.059	0.001	0.054	10.384***
	(0.040)	(0.048)	(0.022)	(0.053)	(3.637)
Mean of dependent variable	0.39	0.58	0.95	0.56	18.66
Observations	11,120	12,651	12,694	12,250	11,029

Notes: The data used are from the HRS 1998 to 2014 of individuals who are potentially in need of LTC and eligible for MAWs. Each cell reports estimates using a TWFE model with state and year FEs for each dependent variable. All dependent variables are outcomes in the previous two years. Home health use in column 1 is an indicator showing that an individual used paid home health use; hospital use in column 2 represents whether an individual visited a hospital; medication in column 3 shows whether an individual regularly took medication; hospital overnight in column 4 indicates whether an individual stayed in a hospital for at least one night; and number of doctor visits in column 5 shows the times that an individual paid for a doctor visit. All models control for state FEs, year FEs, and state-specific linear time trends. Standard errors are clustered at the state level

***p < 0.01

Table 8 represents the channel of MAWs on healthy behaviors such as drinking and smoking. The MAW program significantly reduces the likelihood of smoking now by 5 percentage points, which is about 67 percent reduction with a 8 percent of older individuals smoking. MAWs do not have effects on ever drinking or ever smoking as shown in columns 1 and 4, which is expected since long-lasting drinking and smoking behavior is hard to change by persuasion of providers. However, the drinking frequencies per day or per number a day is decreased some while the magnitude of the reduction is small.

**Table 8:** Channel of MAWs on behavior of drinking and smoking

	(1)	(2)	(3)	(4)	(5)
	Drink ever	Drink days	Drink number per day	Smoke ever	Smoke now
MAW expenditures	0.009	-0.024	-0.007	0.025	-0.054**
	(0.027)	(0.109)	(0.075)	(0.058)	(0.021)
Mean of dependent variable	0.20	0.34	0.18	0.54	0.08
Observations	12,717	12,697	12,688	12,588	12,631

Notes: The data used are from the HRS 1998 to 2014 of individuals who are potentially in need of LTC and eligible for MAWs. Each cell reports estimates using a TWFE model with state and year FEs for each dependent variable. All dependent variables are outcomes in the previous two years. Drink ever in column 1 is an indicator of whether respondents have ever drank alcohol; Drink days in column 2 indicates the number of days per week respondents have had any alcohol to drink in the last three months, for example, beer, wine, or any drink containing liquor; Drink number per day in column 3 shows the number of drinks per day respondents have consumed in the last three months on the days they have been drinking; Smoke ever in column 4 is a dichotomous indicator of whether respondents have ever smoked; and Smoke now is a dichotomous indicator of whether respondents were smoking at the time of being surveyed. All models control for state FEs, year FEs, and state-specific linear time trends. Standard errors are clustered at the state level

**p < 0.05

## Conclusion

In this paper, I explore how the MAW program is associated with health outcomes among older people who are at risk of requiring LTC needs and who are potentially resources constrained to be eligible for Medicaid. Using the within-individual variation of MAW spending over years in the period 1998–2014, the results show that the program is beneficial to improve health outcomes for older people aging at home or in their communities. Specifically, an increase in MAW spending is significantly correlated with a reduction in the probability of self-reporting poor health, mobility/IADL limitations, and depression. The improving effect of MAWs on health is supported by the placebo tests that individuals who have high income or who possess valuable assets or who are younger and healthy are not affected by the program.

The findings of this study have several policy implications. First, the results are informative for the development of LTC policy. During the 2020 pandemic, CMS changed the implementation rules of MAWs and were permitted to loosen quality requirements for home health care providers in order to ensure that services would continue to be provided to the participants. In addition, some states increased payment rates in order to attract more providers and to compensate providers for the increased risk of entering homes during the crisis. Understanding the detailed effects of MAWs on health outcomes is essential, as the federal government is planning for the eventual return to regular operations. The results can help inform policy debates about increasing government investment in home-based programs that meet older people’s preference to age in place and also help justify the $400 billion expansion of the American Jobs Plan to increase HCBS coverage by the Biden Administration at the end of March 2021.

In addition, improving the quality of care provided by home health agencies is a leading priority of CMS while reducing costs by shifting LTC resources to home- or community-based settings. While each state MAW program has minimum requirements for the certification of LTC providers that are regulated by the federal government, these requirements can be difficult to monitor and vary significantly across states. Moreover, states are responsible for surveying and monitoring home health agencies to ensure that they provide services with high quality. However, with so many individuals being served by thousands of agencies, it is difficult to monitor each service or each provider, thus to ensure that all patients are treated fairly. The findings in this paper provide direct evidence on health effects of MAWs, which can be discussed in depth, and be used to create value-based quality indicators to more efficiently regulate home health care providers.

While this study provides valuable insights into the relationship between state-level MAW spending and health outcomes for older adults using observational data from the HRS, there are notable limitations to consider. Observational studies cannot definitively establish causal relationships. There might be other unobserved factors, such as socioeconomic status, access to healthcare services, regional health infrastructure, and lifestyle choices, that influence both MAW spending and health outcomes, potentially biasing the results. Although placebo tests were conducted to address some of these concerns, they do not entirely eliminate the possibility of confounding variables. Consequently, the findings should be interpreted with caution, acknowledging the inherent limitations of the observational study design.

### Supplementary Information

Supplementary Material 1.

## Data Availability

The data that support the findings of this study are available from the Health and Retirement Study Survey Research Center, University of Michigan but restrictions apply to the availability of these data, which were used under license for the current study, and so are not publicly available. Data are however available from the approval from the center. The application process is available on the website.

## Footnotes

1 Many OECD countries have shifted resources toward providing more affordable home-based care to reduce the costs of providing long-term institutional care [33].

2 Some of these approaches include Integrated Care Models, Technology-Enabled Care, Person-Centered Care, and Dementia Care.More details on each model can be found on the CMS website at https://www.medicaid.gov/medicaid/waivers/index.html.

3 2014 is the recent year that has consistent policy variables publicized by the CMS.

4 The annual expenditures data can be downloaded at https://www.medicaid.gov/. The enrollment report is collected by Kaiser Family Foundation and publicly available on its website.

5 Such as percentage of population below the poverty level, percentage married, percentage female, education level, percentage white, as well as the size of the total population and population aged 65+.

6 See Liu and Zai [35] for detailed description of these variables.

7 I use total income of respondents which includes pensions and annuities; earnings of their wage or salary income, bonuses or overtime pay or commissions or tips, second job or military reserve earnings, professional practice or trade income; income from Social Security disability (SDI) and Supplemental Security Income (SSI); income from their Social Security retirement, spouse or widow benefits; income from unemployment and worker’s compensation; and income from other government transfers such as veterans’ benefits, welfare, and food stamps. The household asset value is the net value of total assets minus total debts. The value of total assets includes net value of businesses; net value of IRA; net value of stocks, mutual funds, and investment trusts; value of checking, savings, or money market accounts; value of CDs, government savings bonds, and treasury bills; net value of bonds or bond funds; and net value of all other savings.

8 I start from 1998, because I am using the average of policy spending in 1997 and 1998 as the treatment value in HRS survey year 1998. See section "Estimation" for details.

9 The same procedure is also employed in Dave et al. [12] using the HRS, in Eibich [15] using the German Socio-Economic Panel Study (SOEP), and in Kuka [32] using the Survey of Income and Program Participation (SIPP).

10 More details on the construction of these measures can be found in Chien et al. [9]. Full results are available upon request.

11 The results on each health outcome with full list of cutoffs are available upon request. The Online Table selects some alternative health outcomes to report to save space.

12 For the sample of individuals who are potentially eligible for Medicaid and who are likely to be dependent, they are plausibly start to have helpers and be on some healthcare service when observed in the HRS so a TWFE model with a state-year panel is used to increase the variations from MAW expenditure.
