# Supplementary material for: Evaluating the health outcomes of aging in place: the role of medicaid aging waiver program on U.S. older adults
Source: BMC Public Health. 2024 Aug 5;24:2104. doi: 10.1186/s12889-024-19498-3 (PMC11299275; doi:10.1186/s12889-024-19498-3)
Supplement: Supplementary file 1 — Supplementary Material 1. [file 12889_2024_19498_MOESM1_ESM.pdf]

Table A1: Relationship Between HCBS in 1998 and Pre-Expansion Health Variables

Dependent Variable	(1)<br>Mean in 1998	(2)<br>Univariate<br>HCBS in 1998	(3)<br>Multivariate<br>HCBS in 1998
	Panel A: Health outcomes (1992-1998)		
Poor health status (0-1)	0.315	-0.046**	-0.017
		(0.019)	(0.240)
Mobility limitation (0-1)	0.264	-0.016	0.542*
		(0.035)	(0.271)
ADL limitation (0-1)	0.092	-0.010	0.424
		(0.029)	(0.253)
IADL limitation (0-1)	0.080	0.062	-0.007
		(0.054)	(0.396)
Mental depression (0-1)	0.066	0.003	0.089
		(0.015)	(0.162)
	Panel B: Health care use (1992-1998)		
Drugs	0.728	-0.049	-0.042
		(0.045)	(0.308)
Doctor visit	0.932	-0.012	-0.054
		(0.033)	(0.171)
Nurse home stay	0.033	-0.005	0.046
		(0.014)	(0.160)
Dentist visit	0.619	0.175***	-0.444
		(0.050)	(0.357)

Notes: The data used are from HRS 1992-1998 of individuals who are aged 65 and older. The HCBS in 1998 is per capita spending of older population. The first column reports the mean of each dependent variable. Column 2 estimates the univariate relationship between HCBS in 1998 and health outcomes. Column 3 estimates the multivariate version with controls from specification (1). The univariate regression is a simple model of specification (1) without any controls. The multivariate regression adds demographics of individuals such as age, age squared, the number of living siblings, and marital status. Standard errors are clustered at the state level and shown in parentheses. *** p<0.01, ** p<0.05, * p<0.10.

Table A2: Effect of State Economic Conditions on HCBS

	Dependent Variable: HCBS Expenditure in 1998							
	(1)	(2)	(3)	(4)	(5)	(6)	(7)	(8)
Unemployment rate	-19.815**	-36.451			-71.490	-79.548	-63.966	-80.971
	(8.442)	(49.515)			(56.412)	(59.791)	(59.326)	(64.207)
Unemployment rate^2		0.325			5.116	5.710	4.206	5.970
		(6.585)			(7.705)	(7.480)	(7.698)	(7.310)
Unemployment rate^3		0.051			-0.116	-0.132	-0.079	-0.144
		(0.303)			(0.341)	(0.323)	(0.330)	(0.294)
Employment rate			15.043*	-838	-1,026	-1,142	-1,072	-1,095
			(8.084)	(778)	(825)	(882)	(888)	(882)
Employment rate^2				14.551	17.879	19.801	18.594	19.040
				(12.896)	(13.767)	(14.701)	(14.744)	(14.655)
Employment rate^3				-0.082	-0.102	-0.112	-0.106	-0.108
				(0.071)	(0.077)	(0.082)	(0.081)	(0.081)
GDP per capita					-6,037			-3,375
					(5,418)			(7,897)
PI per capita						-0.012		-0.010
						(0.013)		(0.026)
PCE per capita							-0.010	0.004
							(0.035)	(0.053)
Observations	816	816	816	816	816	816	816	816
Adjusted R-squared	0.958	0.958	0.958	0.958	0.958	0.958	0.958	0.958

Notes: The data used are a state-year panel from 1999 to 2014. The unemployment and employment level is from BLS, the state population is from Census Bureau, the GDP, personal income (PI), personal consumption expenditure (PCE) is from the Bureau of Economic Analysis Regional Analysis Accounts. The dependent variable is per capita HCBS spending. Each cell reports estimates from a separate specification. All regressions include state, year fixed effects and weighted using the state population. Standard errors are clustered at the state level and shown in parentheses. *** p<0.01, ** p<0.05, * p<0.10.

Table A3: Effect of Lagged State Economic Conditions on HCBS

	Dependent Variable: HCBS Expenditure in 1998								
	(1)	(2)	(3)	(4)	(5)	(6)	(7)	(8)	(9)
Unemployment rate lag 1	-32.093**	-22.273							-25.561
	(12.755)	(64.547)							(51.984)
Unemployment rate lag 1^2		-2.339							-1.210
		(9.436)							(7.689)
Unemployment rate lag 1^3		0.131							0.086
		(0.386)							(0.333)
Unemployment rate lag 2			-29.028	-47.526					-95.720
			(18.049)	(72.417)					(83.220)
Unemployment rate lag 2^2				3.088					11.088
				(10.269)					(11.517)
Unemployment rate lag 2^3				-0.147					-0.441
				(0.423)					(0.445)
Employment rate lag 1					13.761	-1,084			-1,350
					(10.654)	(1,046)			(973)
Employment rate lag 1^2						18.843			21.319
						(17.244)			(16.216)
Employment rate lag 1^3						-0.107			-0.112
						(0.095)			(0.090)
Employment rate lag 2							10.705	-241.748	-18.742
							(10.606)	(1,273)	(1,090)
Employment rate lag 2^2								5.969	2.870
								(20.352)	(17.386)
Employment rate lag 2^3								-0.042	-0.029
								(0.108)	(0.093)
Observations	765	765	714	714	765	765	714	714	714
Adjusted R-squared	0.961	0.961	0.965	0.965	0.960	0.960	0.964	0.965	0.966

Notes: The data used are a state-year panel from 1999 to 2014. The unemployment and employment level is from BLS, the state population is from Census Bureau, the GDP, personal income (PI), personal consumption expenditure (PCE) is from the Bureau of Economic Analysis Regional Analysis Accounts. The dependent variable is per capita HCBS spending. Each cell reports estimates from a separate specification. The last column includes lagged income controls such as GDP per capita, PI per capita, and PCE per capita. All regressions include state, year fixed effects and weighted using the state population. Standard errors are clustered at the state level and shown in parentheses. *** p<0.01, ** p<0.05, * p<0.10.

Table A4: Effect of HCBS on Other Spending

	(1)	(2)	(3)
	Health care per capita	Net health insurance per capita	Life insurance per capita
HCBS per capita ($1,000)	-0.001	-0.001	0.001
	(0.005)	(0.001)	(0.001)
Observations	816	816	816
Adjusted R-squared	0.997	0.973	0.953
Mean Y	471.9	40.28	24.15
Mean HCBS	498.98	498.98	498.98

Notes: The data used are a state-year panel from 1999 to 2014. The health care, net health insurance, and life insurance spending is from the Bureau of Economic Analysis Regional Analysis Accounts. All regressions include state, year fixed effects and weighted using the state population. Standard errors are clustered at the state level and shown in parentheses. *** p<0.01, ** p<0.05, * p<0.10.

Table A5: The Associations Between MAWs with Health Using Other Cutoffs

	(1)	(2)	(3)
	Panel A: Self-reported Health		
	Main	Poor	
MAW expenditures per person ($1,000)	-0.109*	-0.078	
	(0.061)	(0.082)	
Mean of dependent variable	0.76	0.41	
	Panel B: Mobility Limitation		
	Main	≥ 4	$= 5$
MAW expenditures per person ($1,000)	-0.141***	-0.243**	-0.174***
	(0.035)	(0.091)	(0.060)
Mean of dependent variable	0.92	0.69	0.50
	Panel C: IADL Limitation		
	Main	≥ 2	≥ 4
MAW expenditures per person ($1,000)	-0.141***	-0.078	-0.047
	(0.041)	(0.061)	(0.071)
Mean of dependent variable	0.82	0.66	0.37
	Panel D: Depression		
	Main	≥ 2	≥ 6
MAW expenditures per person ($1,000)	-0.145***	-0.143	-0.052
	(0.053)	(0.104)	(0.085)
Mean of dependent variable	0.89	0.74	0.23

Notes: The data used are from HRS 1998 to 2014 of individuals who are potentially in need of LTC and eligible for MAWs. Each cell reports estimates using equation (1) for each health outcome with different cutoffs. Poor health (main in Panel A) is an indicator showing that an individual self-assesses his or her general health status as fair or poor. Mobility limitation (main in Panel B) is an indicator of having at least 2 items of limitations in walking 1 block, walking several blocks, walking across a room, climbing 1 flight of stairs, and climbing several flights of stairs. IADL limitation (main in Panel C) is an indicator of having at least 1 item of limitations in using the phone, managing money, taking medications, shopping for groceries, and preparing hot meals. Depression (main in Panel D) is an indicator of feeling at least 1 negative emotions in the past two years such as felt depressed, felt alone, felt sad, had restless sleep, felt everything was an effort, could not get going, felt unhappy, and did not enjoy life. All models control for individual FEs, year FEs, state-specific linear time trends, and demographics of individuals such as age, age squared, marital status, and number of siblings. Standard errors are clustered at the state level. *** p<0.01, ** p<0.05, * p<0.10.

Table A6: The Associations Between MAW Expenditures With Other Health Outcomes

	(1)	(2)	(3)	(4)	(5)	(6)	(7)
	Gross	Fine Ability	Large Muscle	Cancer	Lung	Heart	Stroke
MAW per person ($1,000)	-0.050	0.029	-0.038	0.026	-0.031	-0.049	-0.089
	(0.047)	(0.068)	(0.030)	(0.066)	(0.082)	(0.135)	(0.056)
Mean of dependent variable	0.92	0.52	0.92	0.20	0.24	0.57	0.35
Number of individuals	3,124	3,124	3,114	3,118	3,116	3,116	3,116
Observations	9,321	9,321	9,284	9,295	9,292	9,284	9,293
Adjusted R-squared	0.22	0.34	0.16	0.48	0.41	0.33	0.50

Notes: The data used are from HRS 1998 to 2014 of individuals who are potentially in need of LTC and eligible for MAWs. Each cell reports estimates using equation (1) for each dependent variable. Gross motor skills limitation in column 1 includes items of walking one block, walking across a room, climbing one flight of stairs, getting in or out of bed, and bathing activities. Fine motor skills limitation in column 2 includes items of picking up a dime, eating, and dressing activities. Large muscle limitation in column 3 includes items of sitting for 2 hrs, getting up from a chair, stooping, kneeling or crouching, and pushing or pulling large objects activities. Columns 4 to 7 are dichotomous dependent variables indicating whether individuals have been diagnosed with cancer, lung, heart disease, and stroke. All models control for individual FEs, year FEs, state-specific linear time trends, and demographics of individuals such as age, age squared, marital status, and number of siblings. Standard errors are clustered at the state level. *** p<0.01, ** p<0.05, * p<0.10.

Table A7: Robustness Checks of the Results of MAWs on Health with State Controls

	(1)	(2)	(3)	(4)
	Poor health	Mobility limitation	IADL limitation	Depression
MAW expenditures per person ($1,000)	-0.123	-0.151***	-0.208***	-0.176***
	(0.088)	(0.045)	(0.063)	(0.056)
State controls	Y	Y	Y	Y
Mean of dependent variable	0.76	0.93	0.82	0.89
Number of individuals	2,836	2,820	2,835	1,722
Observations	8,335	8,299	8,346	4,984
Adjusted R-squared	0.32	0.21	0.33	0.32

Notes: The data used are from HRS 1998 to 2014 of individuals who are potentially in need of LTC and eligible for MAWs. Each cell reports estimates using equation (1) for each dependent variable. Poor health is an indicator showing that an individual self-assesses his or her general health status as fair or poor. Mobility limitation is an indicator of having at least 2 items of limitations in walking 1 block, walking several blocks, walking across a room, climbing 1 flight of stairs, and climbing several flights of stairs. IADL limitation is an indicator of having at least 1 item of limitations in using the phone, managing money, taking medications, shopping for groceries, and preparing hot meals. Depression is an indicator of feeling at least 1 negative emotions in the past two years such as felt depressed, felt alone, felt sad, had restless sleep, felt everything was an effort, could not get going, felt unhappy, and did not enjoy life. All models control for individual FEs, year FEs, state-specific linear time trends, demographics of individuals such as age, age squared, marital status, and number of siblings, and state-level socioeconomic variables such as percentage below the poverty level, education level, percentage female, percentage white, percentage married, personal income per capita, employment rate, and political affiliation of the governor. Standard errors are clustered at the state level. *** $p<0.01$, ** $p<0.05$, * $p<0.10$.

Table A8: Robustness Checks of the Results of MAWs on Health Dropping Moving Observations

	(1)	(2)	(3)	(4)
	Poor health	Mobility limitation	IADL limitation	Depression
MAW expenditures per person ($1,000)	-0.087*	-0.139***	-0.136***	-0.165***
	(0.051)	(0.030)	(0.035)	(0.040)
Mean of dependent variable	0.76	0.92	0.82	0.89
Number of individuals	2,993	2,981	2,993	1,817
Observations	8,856	8,821	8,867	5,263
Adjusted R-squared	0.32	0.20	0.32	0.29

Notes: The data used are from HRS 1998 to 2014 of individuals who are potentially in need of LTC and eligible for MAWs and who do not migrate over years. Each cell reports estimates using equation (1) for each dependent variable. Poor health is an indicator showing that an individual self-assesses his or her general health status as fair or poor. Mobility limitation is an indicator of having at least 2 items of limitations in walking 1 block, walking several blocks, walking across a room, climbing 1 flight of stairs, and climbing several flights of stairs. IADL limitation is an indicator of having at least 1 item of limitations in using the phone, managing money, taking medications, shopping for groceries, and preparing hot meals. Depression is an indicator of feeling at least 1 negative emotions in the past two years such as felt depressed, felt alone, felt sad, had restless sleep, felt everything was an effort, could not get going, felt unhappy, and did not enjoy life. All models control for individual FEs, year FEs, state-specific linear time trends, and demographics of individuals such as age, age squared, marital status, and number of siblings. Standard errors are clustered at the state level. *** $p<0.01$, ** $p<0.05$, * $p<0.10$.
